# Effects of Heat and Cold—How to Kill Bacteria

**Published:** 1890-11

**Authors:** 


### Effects of Heat and Cold—How to Kill Bacteria.

Within certain limits heat increases and cold retards their growth. Different species differ in regard to the temperature at which they grow best. Most species find the best conditions for growth at temperatures between 70° and 100° F. A few will grow quite readily at a temperature below 50° F. A temperature below freezing stops their growth. The heat of boiling water will kill the active forms, but several successive boilings are required to kill their spores (corresponding to seed). It is not difficult to destroy bacteria after they have once succeeded in getting into the milk. This may be easily done by heating the milk to the boiling temperature for ten minutes upon three successive days. Milk thus deprived of bacteria is said to be sterilized, and if we prevent the further entrance of bacteria by closing the vessel tightly with a plug of cotton it will remain sweet indefinitely.—H. W. Conn, in *Dental Advertiser.*

